# Lipidomics revealed alterations in glycerophospholipid metabolism in skin squamous cell carcinoma

**DOI:** 10.3389/fmolb.2024.1356043

**Published:** 2024-07-23

**Authors:** Li-Hong Mei, Hui-Hui Gan, Hong-Feng Wang, Guoxiong Xu, Xuan-Guang Ye, Gao Yang

**Affiliations:** ^1^ Department of Dermatology, Jinshan Hospital, Fudan University, Shanghai, China; ^2^ Department of Central Laboratory, Jinshan Hospital, Fudan University, Shanghai, China; ^3^ Department of Pathology, Jinshan Hospital, Fudan University, Shanghai, China

**Keywords:** skin squamous cell carcinoma, lipidomics, glycerophospholipid metabolism, biomarkers, therapeutic targets

## Abstract

**Background:**

Skin squamous cell carcinoma (SCC) is a prevalent malignancy, and dysregulated lipid metabolism has been implicated in its pathogenesis. However, detailed characterization of lipid alterations in SCC remains limited.

**Methods:**

We analyzed lipid metabolic variations in tissue samples from 34 SCC patients and adjacent healthy tissues (located more than 1 cm from the tumor margin) using liquid chromatography-mass spectrometry (LC-MS). Data visualization and discriminatory lipid profiles were identified using principal component analysis (PCA) and sparse partial least squares discriminant analysis (sPLS-DA). Key lipids involved in the SCC metabolism were identified and further validated using an external data set (from a previous study, which similarly explored lipid profiles in oral SCC using lipidomics approaches). Pathway enrichment analysis was conducted to elucidate the metabolic pathways associated with these key lipids.

**Results:**

Eight lipids were identified by comparing SCC and healthy tissues including PI(16:0/22:4), PI(18:1/20:4), PE(16:0/20:4), PE(16:0/22:5), PE(16:0/22:6), PE(18:1/20:3), PC(18:1/20:2), and PC(18:2/20:2), as confirmed by independent datasets. All of these lipids were upregulated in SCC tumor tissues. Pathway enrichment analysis revealed significant alterations in glycerophospholipid metabolic pathways, particularly affecting the metabolism of diacylglycerophosphocholines, glycerophosphoethanolamines, and glycerophosphoinositols.

**Conclusion:**

Our findings reveal that dysregulated glycerophospholipid metabolism plays a pivotal role in the development of SCC.

## 1 Introduction

Skin non-melanoma cancer represents a significant global public health burden, with squamous cell carcinoma (SCC) being the second most prevalent type (Wysong, 2023). While surgical resection remains the primary treatment option, recent years have witnessed the emergence of non-surgical modalities like photodynamic therapy (PDT) (Girardi et al., 2021). This promising treatment approach utilizes a photosensitizing compound (5-aminolevulinic acid) to target tumor tissues and induce photochemical reactions through visible light irradiation. However, PDT efficacy is often limited by the inability of the photosensitizer to penetrate deep SCC tissues due to the presence of excessive solid lipids and liposomes (Juszczak et al., 2022; Souto et al., 2022). Additionally, elevated lipid concentrations in the cytoplasmic membrane have been linked to altered activity of death receptors in SCC cells (Fecker et al., 2007).

Emerging evidence suggests that dysregulated lipid metabolism plays a crucial role in SCC pathogenesis (Zeng et al., 2021). The altered lipid metabolism is characterized by increased fatty acid, cholesterol, and inositol biosynthesis (Zeng et al., 2021). This dysregulation in lipid homeostasis contributes to membrane biogenesis, energy storage, and signaling pathways that promote tumorigenesis (Celis Ramírez et al., 2020). SCC cells also demonstrate increased demand for specific amino acids, such as glutamine and alanine, to support protein synthesis and other cellular processes (Mei et al., 2022). This altered amino acid profile presents potential targets for therapeutic intervention. Understanding the regulation of lipids and amino acids metabolism could lead to the development of drugs that target these pathways. However, comprehensive profiling of lipid metabolism in SCC remains limited.

Lipidomics, a powerful analytical technique, enables the identification and quantification of individual lipid species, providing valuable insights into cellular and tissue lipid metabolism (Züllig et al., 2020). By comparing lipid profiles across different physiological states, lipidomics allows for the identification of key lipid biomarkers and elucidates the mechanistic roles of lipids in diverse biological processes (Li et al., 2022). Studies employing lipidomics have identified specific lipid species that are upregulated or downregulated in SCC compared to healthy tissues (Zhao et al., 2018). For example, increased levels of sphingolipids and ceramides have been associated with SCC aggressiveness (Sugihara et al., 2018). Additionally, altered glycerophospholipid profiles have been linked to altered membrane dynamics and signaling in SCC cells (Putzhammer et al., 2016). Although spatial metabolomics could provide insights into the localization of metabolites within tissues, its complexity and lower throughput, and the study’s focus on lipid identification and quantification made lipidomics a more suitable and efficient choice.

In this study, we employed liquid chromatography-mass spectrometry (LC-MS) to analyze the lipidomics profile of tumor tissues obtained from SCC patients. LC-MS is effective in detecting a wide range of lipid species and quantifying their levels with high sensitivity, specificity, and comprehensive lipid profiling capabilities, which is essential for identifying biomarkers and understanding lipid metabolism in SCC (Zeng et al., 2021). The choice aligned with the study’s aim to profile and compare lipid compositions between SCC and healthy tissues, offering robust and reproducible results crucial for SCC research. Our primary objective was to identify key lipid species involved in SCC metabolism and contribute to a deeper understanding of the role of altered lipid homeostasis in this malignancy.

## 2 Materials and methods

### 2.1 Ethics

This retrospective cohort study was conducted in accordance with the Declaration of Helsinki. This study was reviewed and approved by the Institutional Review Board of Jinshan Hospital (JIEC 2023-S85). Written informed consent was obtained from all participants prior to enrollment for publication of any potentially identifiable data or images.

### 2.2 Patient population and inclusion/exclusion criteria

From October 2011 to October 2023, the skin tumor sample library was searched. The pathological and clinical information of adult patients undergoing surgical resection for skin tumors at Jinshan Hospital was retrieved in the study. Inclusion criteria were: 1) Histopathologically confirmed SCC; 2) Age ≥18 years. Exclusion criteria included: 1) Presence of systemic infection; 2) Incomplete clinical data; 3) Loss to follow-up. The patients were followed for up to 1 year to monitor tumor recurrence and metastasis. Finally, 34 patients with SCC were enrolled.

### 2.3 Clinical data collection and sample acquisition

Demographic and clinical data (age, gender, tumor location) were collected for all participants. During surgery, both the SCC tumor and control tissue (healthy tissue ≥1 cm from the tumor margin) were excised for histopathological examination and subsequent lipidomic analysis. Tissue samples were frozen and stored at −80°C until analysis.

### 2.4 Chemicals, sample preparation and lipidomics analysis

Isopropanol was used for initial sample preparation, while chloroform and methanol were part of the internal standard mixture. The lipid internal standards included species within the classes of cholesteryl ester (CE(18:1), CE(20:4), CE(22:6)), diacylglycerol (DAG(16:0/16:0), DAG(16:0/18:1), DAG(18:0/18:1), DAG(18:0/22:6)), lysophosphatidylcholine (LPC(16:0)), lysophosphatidylethanolamine (LPE(18:0)), phosphatidylcholine (PC(14:0/14:0)), phosphatidylethanolamine (PE(18:0/18:0), PE(18:0/22:6)), phosphatidylinositols (PI(16:0/16:0)), sphingomyelin (SM(16:0), SM(18:1), SM(24:1)), and triacylglycerol (TAG((16:0/18:1/18:1), TAG((16:0/18:1/22:6), TAG((18:0/18:1/18:2), TAG((18:1/18:1/18:2)). The mobile phases for LC-MS analysis included ammonium acetate and acetonitrile, with nitrogen gas aiding the electrospray ionization process. All reagents were sourced from Sigma-Aldrich and Thermo Fisher Scientific.

Tissue samples were prepared by adding 350 µL isopropanol and 9 µL internal standard mixture (A cold chloroform:methanol = 2:1 solution containing 1 μM LM6002), pre-cooled at 4°C, then mixed for 1 min. After 10 min of incubation at 24°C, samples were stored overnight at −20°C, followed by centrifugation at 12,000 g for 20 min. The supernatant was collected for LC-MS analysis. A targeted lipidomics approach was applied to screen the lipids (Dickinson et al., 2020).

Lipidomic analysis was performed using an AB SCIEX QTRAP 5500 system equipped with a Waters Acquity UPLC BEH HILIC VanGuard pre-column. Mobile phases were composed of acetonitrile-water mixtures with ammonium acetate. Electrospray ionization (ESI) in both positive and negative ion modes was employed for data acquisition.

The samples were injected into a Pre-column (2.1 mm × 5 mm, 1.7 μm, Waters Acquity UPLC BEH HILIC VanGuard). The phase A contained 95% acetonitrile (acetonitrile:water, 95:5) and 10 mmol/L of ammonium acetate. The phase B contained 50% acetonitrile (acetonitrile:water, 50:50) and 10 mmol/L of ammonium acetate. Ammonium hydroxide was added to phase B until its pH was equal to the pH of phase A. The flow rate was set to 0.5 mL/min. The elution gradient was as follows: Phase B started at 0.1%, raised to 20% in 10 min, then raised to 98% in the next 10–11 min, and remained for 2 min, and then returned to the initial 0.1%, which maintained until 16 min. Nitrogen was used as the solvent in the electrospray ionization process. Injection volumes were set to 2 μL for positive mode (ESI+) and 5 μL for negative mode (ESI-). The parameters were set as follows: curtain gas was 35 psi, GS1 was 50 psi, GS2 was 60 psi, ion spray voltage was 5,500 V, declustering potential was 80 V, entrance energy was 10 V, and collision energy collision energy was 50 V.

### 2.5 Data processing

Raw LC-MS data were processed in Analyst software (version 1.7, SCIEX, MA, United States) for peak extraction and alignment. The data, including specific m/z values and fragmentation patterns, were analyzed to determine the composition and positional specificity of the fatty acids in phospholipids. MultiQuant software (AB SCIEX) facilitated lipid identification and quantification, referencing the LIPID MAPS database. The assignment to the sn1 position and the sn2 position is based on the known fragmentation behavior of molecules. Lipids detected in at least 80% of samples in either group were included, with missing values replaced by the median. Data normalization was performed using Pareto scaling prior to performing principal component analysis (PCA) and sparse partial least squares discriminant analysis (sPLS-DA) for data visualization and identification of discriminatory lipid profiles. Pooled QC samples were prepared by combining aliquots from all individual samples to create a representative mix. These pooled QC samples were used to monitor the performance of the LC-MS system throughout the analysis.

### 2.6 Selection and validation of key lipids

Key lipids were identified using stringent selection criteria to ensure their significance and reliability. These criteria included a variable influence on projection (VIP) score greater than 1, indicating the importance of the variable in the model, a false discovery rate (FDR) less than 0.05, ensuring statistical significance, and an area under the receiver operating characteristic curve (AUC) greater than 0.8, reflecting strong discriminative power. To validate these findings, the same criteria were applied to a publicly available lipidomics dataset from a study by Dickinson et al., which compared lipid profiles between 9 oral SCC tumor tissues and 10 healthy tissues using LC-MS (Züllig et al., 2020). Lipids that consistently met these criteria in both our dataset and Dickinson et al.'s dataset were defined as the key lipids associated with SCC tumor tissues, demonstrating their robust association with the disease.

### 2.7 Pathway enrichment analysis

Key differentially expressed lipids were first queried against the Kyoto Encyclopedia of Genes and Genomes (KEGG) database (https://www.kegg.jp/) to predict potential metabolic pathways affected by altered lipid levels in SCC. MetaboAnalyst (https://www.metaboanalyst.ca/) was used to identify significantly altered metabolic pathways associated with SCC lipid metabolism.

### 2.8 Statistical analysis

Statistical analyses were conducted using R (V4.3.2) software. Data were assessed for normality and homogeneity of variance, and either paired t-tests or Kruskal-Wallis H tests were employed for group comparisons based on the results. The Benjamini-Hochberg procedure was utilized for multiple comparison corrections, and a *p*-value <0.05 was considered statistically significant. Results are presented as mean ± standard deviation (SD).

## 3 Results

### 3.1 Patient demographics and clinical characteristics

**FIGURE 1:**
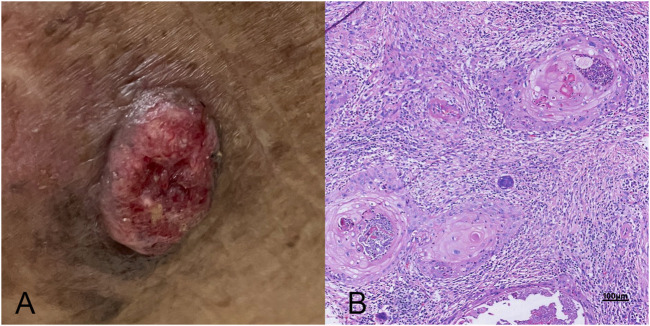
A comprehensive view of skin squamous cell carcinoma (SCC). **(A)** A clinical photograph showing the typical external appearance of SCC, characterized by a raised, scaly, and possibly ulcerated lesion with irregular borders. **(B)** The histopathological examination under high magnification, highlighting the microscopic features of SCC (high power field, 10 × 40), including keratin pearls, atypical keratinocytes with nuclear atypia, and evidence of invasive growth into deeper skin layers.

The study enrolled 34 patients with SCC. Their average age was 72 ± 11.5 years (range: 47–92 years), with no statistically significant difference between males (n = 18; 71 ± 9.4 years; 56–84 years) and females (n = 16; 73 ± 13.0 years; 47–92 years). During the follow-up period, no patients experienced recurrence or metastasis, highlighting the potential effectiveness of the employed therapeutic approach. Detailed clinical characteristics are presented in Table 1, with a representative case illustrated in Figure 1.

**TABLE 1:** Clinical characteristics of the SCC patients.

	Cases (N = 34)
Gender	
Female	16 (47.1%)
Male	18 (52.9%)
Age	71.7 (11.5)
Location	
Left cheek	2 (5.9%)
Left chest	2 (5.9%)
Left face	6 (17.6%)
Left head	5 (14.7%)
Left neck	1 (2.9%)
Left shin	4 (11.8%)
Right check	2 (5.9%)
Right chest	1 (2.9%)
Right face	6 (17.6%)
Right head	2 (5.9%)
Right neck	1 (2.9%)
Right shin	2 (5.9%)

Data presented as N (%) or mean (SD).

### 3.2 Lipidomic profiling, identification of key markers and validation

**FIGURE 2:**
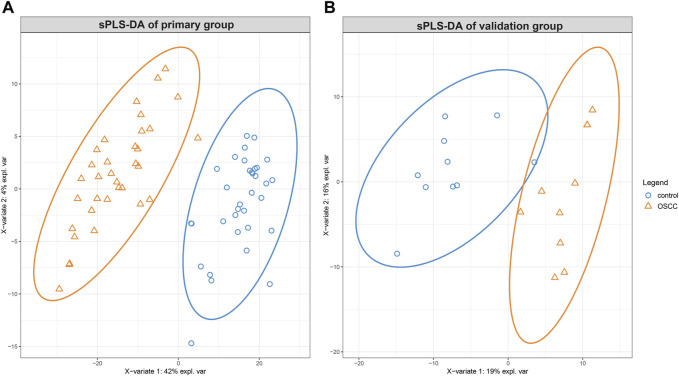
The sparse partial least squares discriminant analysis (sPLS-DA) of lipidomic alterations in skin squamous cell carcinoma (SCC) compared to healthy tissues. Displaying a distinct clustering pattern between SCC and healthy control tissues for the primary group **(A)** and the validation group **(B)**.

Analysis of the primary group revealed a rich lipidomic landscape, with 781 lipids identified initially. After applying stringent data quality criteria, 599 lipids were retained for further analysis. PCA plots indicated differential lipid regulation between groups and QC samples (Supplementary Figure S1). Utilizing sPLS-DA, a powerful statistical technique for dimensionality reduction and group discrimination, these lipids were effectively separated into distinct clusters (Figure 2A). The high predictive power of the model was confirmed by the R2X, R2Y, and Q2 values of 0.46, 0.91, and 0.85, respectively, indicating good model fitness and accurate classification capabilities.

A meticulous selection process identified 65 key lipids based on pre-defined criteria. These key lipids were deemed to be the most informative and reliable markers for discriminating between SCC and control samples.

Similarly, the validation group yielded valuable insights into the lipidomic profile of SCC. From the initial identification of 1370 lipids, 430 were deemed suitable for further analysis. Applying sPLS-DA, the model effectively separated the samples into distinct groups, as demonstrated by the score plot in Figure 2B. While the R2X, R2Y and Q2 values were 0.19, 0.76, and 0.62, respectively.

**FIGURE 3:**
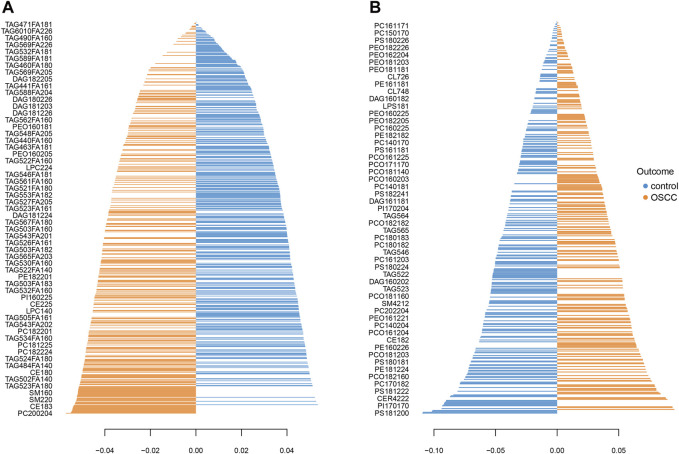
Loading plot shows the lipid profiles of SCC and healthy control tissues in the primary **(A)** and the validation **(B)** group. The blue bars indicate higher levels in control tissues and the orange bars indicate higher levels in SCC.

Loading plots for both datasets (Figure 3) provide detailed information about the contributions of individual lipids to the observed group separation. These plots allow for a deeper understanding of the underlying biological mechanisms and the specific lipid species driving the observed differences between SCC and control samples.

**FIGURE 4:**
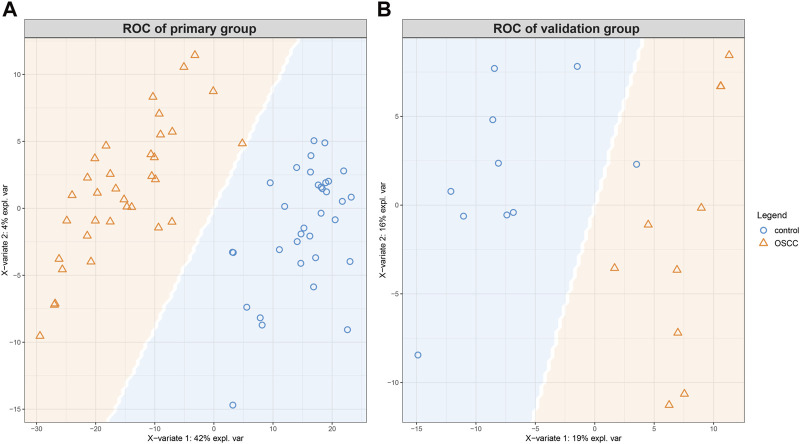
The prediction background plot shows the distribution of data, the presence of biases, or the existence of outliers. The SCCs and control tissues could be classified according to the model generated by sPLS-DA for the primary **(A)** and the validation **(B)** groups.

Furthermore, the prediction background plot (Figure 4) provides a visual representation of how individual samples were classified by the sPLS-DA model in both groups. This visual representation further confirms the effectiveness of the model in accurately discriminating between SCC and control samples.

### 3.3 Consistent identification of key lipids and their upregulation

A comparison of the key lipids identified in the primary and validation groups revealed a high degree of consistency. Eight key lipids consistently demonstrated significant upregulation in SCC tumor tissues compared to control tissues, highlighting their robust association with the disease. These lipids were PI(16:0/22:4), PI(18:1/20:4), PE(16:0/20:4), PE(16:0/22:5), PE(16:0/22:6), PE(18:1/20:3), PC(18:1/20:2), and PC(18:2/20:2). This finding provided evidence for the potential role of these specific lipids in SCC pathogenesis and their potential utility as biomarkers for diagnosis, prognosis, or therapeutic targeting (Table 2).

**TABLE 2:** The influence on projection scores, false discovery rate and receiver operating characteristic curve areas of the key lips in primary and validation groups.

Lipids	Primary cohort				Validation cohort			
	FDR	VIP	AUC	FC	FDR	VIP	AUC	FC
PC(18:1/20:2)	<0.001	1.44	1.00	1.5	0.017	1.67	0.92	1.8
PC(18:2/20:2)	<0.001	1.37	0.99	1.2	0.025	1.57	0.84	2.1
PE(16:0/20:4)	<0.001	1.41	1.00	2.5	0.049	1.33	0.84	2.2
PE(16:0/22:5)	<0.001	1.39	0.99	2.7	0.017	1.67	0.88	2.0
PE(16:0/22:6)	<0.001	1.32	0.97	1.9	0.049	1.34	0.83	2.3
PE(18:1/20:3)	<0.001	1.36	1.00	2.7	0.025	1.58	0.82	1.7
PI(16:0/22:4)	<0.001	1.33	0.98	1.4	0.005	1.97	0.93	2.4
PI(18:1/20:4)	<0.001	1.33	0.97	1.3	0.017	1.67	0.86	1.7

AUC, receiver operating characteristic curve area; FC, fold change; FDR, false discovery rate; VIP, influence on projection.

### 3.4 Pathway enrichment analysis and dysregulated glycerophospholipid metabolism

**FIGURE 5:**
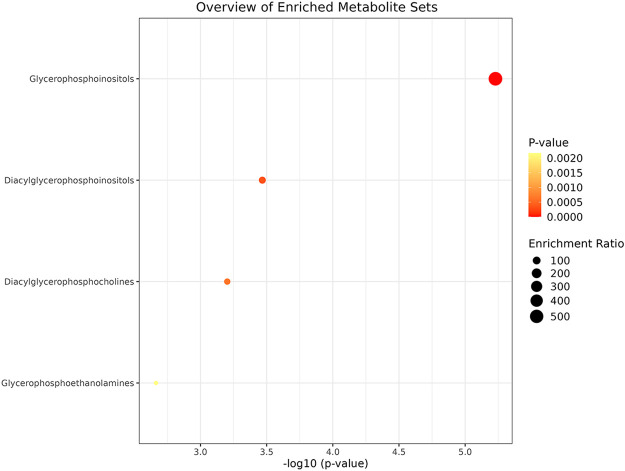
The pathway enrichment analysis for different lipid classes in SCC using a bubble plot. The *x*-axis displays the negative logarithm of the *p*-value, indicating the statistical significance of each metabolite set’s enrichment, while the *y*-axis lists the metabolite sets. Bubble size represents the enrichment ratio, and color intensity (from yellow to red) reflects the *p*-value, with red indicating higher significance. Pathway analysis shows that diacylglycerophosphocholines metabolism, glycerophosphoethanolamines metabolism, glycerophosphoinositols metabolism and diacylglycerophosphoinositols metabolism participate in the lipid metabolism of SCC.

To gain deeper insights into the biological implications of the observed lipidomic changes, pathway enrichment analysis was conducted. This analysis revealed a significant involvement of glycerophospholipid metabolic pathways in SCC. Specifically, alterations were identified in diacylglycerophosphocholines metabolism, glycerophosphoethanolamines metabolism, glycerophosphoinositols metabolism, and diacylglycerophosphoinositols metabolism (Figure 5).

## 4 Discussion

Lipids are essential cellular components with diverse roles in membrane structure, energy storage, cell signaling, and differentiation. Dysregulated lipid metabolism is a hallmark of cancer, and recent studies have implicated it in the pathogenesis of skin SCC (Souto et al., 2022). Our study employed advanced lipidomics techniques to investigate the lipid profiles of SCC tumor tissues and elucidate the potential mechanisms underlying their altered metabolism.

Our findings confirmed the significant upregulation of several glycerophospholipids, including phosphatidylethanolamines (PE), phosphatidylcholines (PC), and phosphatidylinositols (PI), in SCC tissues. This finding aligns with previous research demonstrating increased levels of glycerophospholipids in various cancer types, including oral squamous cell carcinoma (Dickinson et al., 2020). This consistent observation underscores the potential importance of glycerophospholipid metabolism in SCC tumorigenesis and disease progression.

The elevated levels of PC in SCC are particularly noteworthy, as PC accounts for approximately half of the total phospholipid content in cell membranes and plays a crucial role in maintaining membrane structure and function (Wang et al., 2017; Zhi et al., 2021). Previous studies have also demonstrated an association between increased PC levels and the high proliferation rate observed in carcinoma cells (Zeng et al., 2021). Our findings support this notion and suggest that the upregulation of PC in SCC might contribute to its aggressive behavior. PC(16:0/18:1) increase in SCC highlights changes in membrane lipid composition and turnover. While PC(18:0/18:2) elevation suggests metabolic reprogramming in SCC cells to support rapid cell growth and division (Wang et al., 2017; Zhi et al., 2021). Interestingly, some studies investigating PC levels in lung SCC reported contrasting results (Zeng et al., 2021). This discrepancy could be attributed to variations in sample types, testing methods, and tumor stages. Nonetheless, these findings highlight the complex and dynamic nature of lipid metabolism in cancer and emphasize the need for further research to fully understand the role of specific lipids in different cancer types and contexts. Changes in PC levels can influence PS, PE, and PI levels through glycerophospholipid metabolism, suggesting a complex interplay in lipid metabolic pathways (Reed et al., 2021).

The observed upregulation of PE in SCC is also noteworthy, considering its critical roles in cell proliferation, maintaining mitochondrial stability, and regulating energy production (Melo et al., 2016; Kim et al., 2023). PE(16:0/20:4) and PE(18:0/20:4) were significantly elevated in SCC samples. Their role in autophagy and apoptosis regulation is critical in cancer, and their upregulation may reflect the altered membrane dynamics and cellular stress responses in SCC cells. The interconnectedness of PC and PE metabolism suggests that further investigation into the interplay between these two phospholipids could provide valuable insights into the mechanisms underlying SCC development (Infantino et al., 2021).

Elevated PI levels in SCC further support the notion of dysregulated phosphoinositide signaling pathways in cancer. PI is a key component of the endoplasmic reticulum and plays a pivotal role in various cellular processes, including cell survival, metabolism, and growth, particularly through the PI3K/Akt/mTOR signaling pathway (Ersahin et al., 2015). The upregulation of PI in SCC tissues suggests that this pathway might be activated in tumor cells, potentially promoting tumorigenesis. The significant elevation of PI(16:0/22:4) and PI(18:0/18:1) suggests enhanced phosphoinositide signaling, which is known to contribute to cancer cell proliferation and metastasis.

Sphingolipids like ceramide and sphingomyelin also showed variations in SCC, yet they were not identified as key lipids due to the study’s selection and validation methods. Despite this, bioactive sphingolipids are known to regulate tumor cell growth, differentiation, and angiogenesis, as seen in ovarian and breast cancers (Li et al., 2017; He et al., 2022). Sphingomyelin has been reported to activate various signaling cascades and is upregulated in cancer tissues (Taniguchi and Okazaki, 2021). Upregulation of SM(d18:1/16:0) suggests alterations in sphingolipid metabolism, which is crucial for cell growth, differentiation, and apoptosis.

While our study identified PC, PE, and PI as key lipids in SCC, other lipid classes also exhibited differential expression. Notably, we observed a decrease in triacylglycerol (TAG) levels in SCC compared to control tissues. This finding is consistent with a previous study by Dickinson et al. (Dickinson et al., 2020) and suggests that TAG metabolism might be downregulated in SCC (Prendeville and Lynch, 2022). As TAG serves as the primary storage form of lipids and a substrate for PC and PE synthesis, its reduced abundance might reflect increased energy demands and active lipid synthesis within the tumor environment (Cui and Liu, 2020).

Our study’s strength was the use of an external dataset for validation enhances the reliability of our lipidomics results. The identified lipids hold promise as biomarkers for early SCC diagnosis and prognosis. Future research should explore their roles in SCC pathogenesis and potential as therapeutic targets. Combining lipidomics with other omics approaches, like proteomics and transcriptomics, could enhance our understanding of SCC and lead to novel treatments. However, certain limitations must be acknowledged. Discrepancies in LC-MS methods across different studies led to the non-correspondence of some metabolites, which may limit the direct comparison of absolute values. Additionally, the relatively small sample size of our study warrants further validation in larger, multicenter, and prospective studies to confirm the generalizability of our findings.

## 5 Conclusion

Our study unveils dysregulated glycerophospholipid metabolism as a pivotal factor in SCC development.

## Data Availability

The raw data supporting the conclusions of this article will be made available by the authors, without undue reservation.

## Supplementary material

SUPPLEMENTARY FIGURE S1
Principal component analysis (PCA) plot of the lipids with QC samples.
