# Supplementary material for: Lipidomics revealed alterations in glycerophospholipid metabolism in skin squamous cell carcinoma
Source: Front Mol Biosci. 2024 Jul 23;11:1356043. doi: 10.3389/fmolb.2024.1356043 (PMC11300206; doi:10.3389/fmolb.2024.1356043)
Supplement: Supplementary file 1 [file DataSheet1.docx]

Supplementary Material

## Supplementary Figures


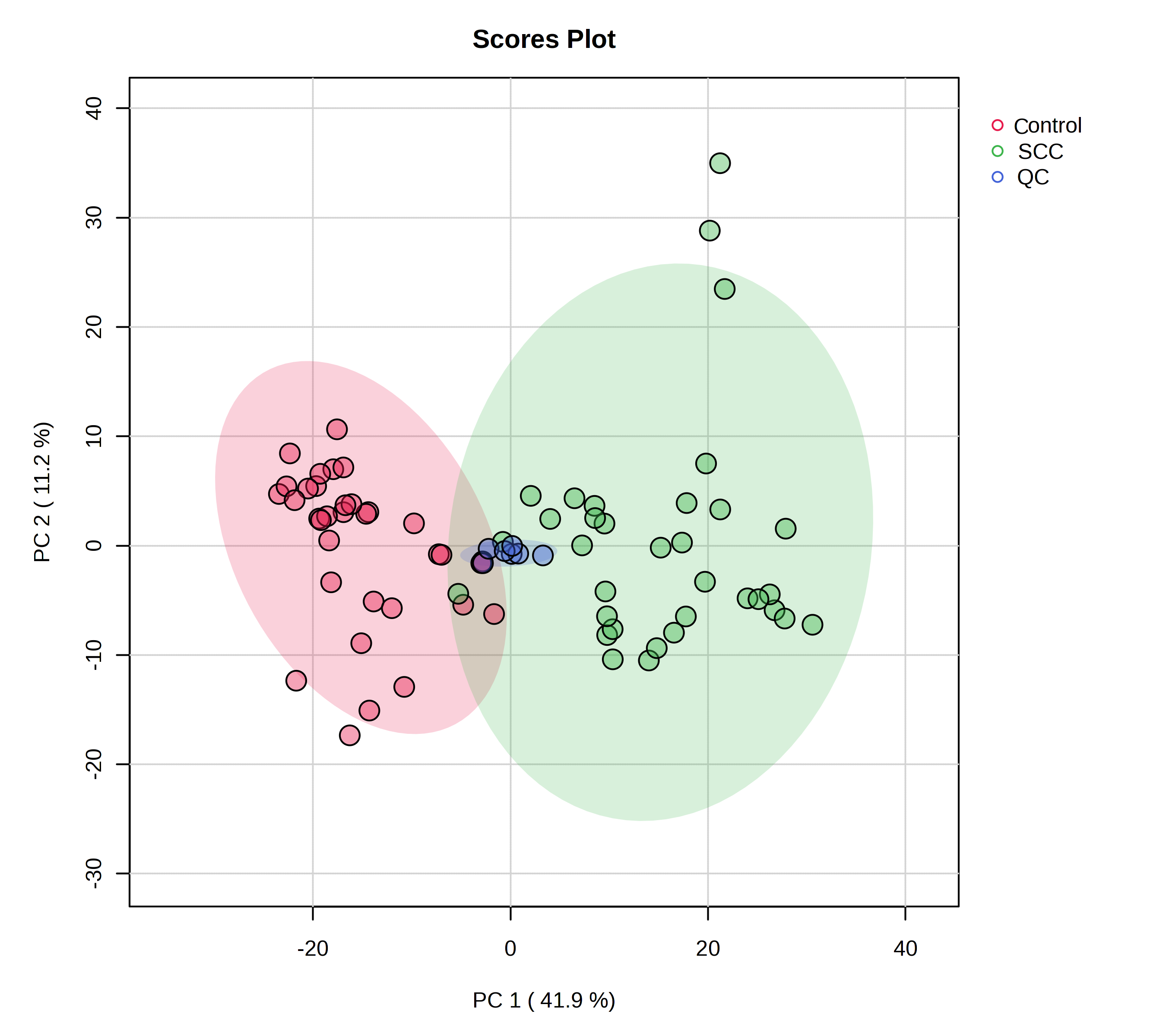


**Supplementary Figure 1.** Principal component analysis (PCA) plot of the lipids with QC samples.

## Detailed Transitions for Lipids Identified in the Study:

1. Lysophosphatidylcholines (LPCs):

LPC(14:0)

Precursor ion: [M+H]⁺ at m/z 468.3

Product ions: 184.1 (Phosphocholine head group), 226.3 (14:0 fatty acid)

LPC(16:0)

Precursor ion: [M+H]⁺ at m/z 496.3

Product ions: 184.1, 256.3 (16:0 fatty acid)

LPC(16:1)

Precursor ion: [M+H]⁺ at m/z 494.3

Product ions: 184.1, 254.3 (16:1 fatty acid)

LPC(18:0)

Precursor ion: [M+H]⁺ at m/z 524.3

Product ions: 184.1, 284.3 (18:0 fatty acid)

LPC(18:1)

Precursor ion: [M+H]⁺ at m/z 522.3

Product ions: 184.1, 282.3 (18:1 fatty acid)

LPC(18:2)

Precursor ion: [M+H]⁺ at m/z 520.3

Product ions: 184.1, 280.3 (18:2 fatty acid)

LPC(18:3)

Precursor ion: [M+H]⁺ at m/z 518.3

Product ions: 184.1, 278.3 (18:3 fatty acid)

LPC(20:0)

Precursor ion: [M+H]⁺ at m/z 552.3

Product ions: 184.1, 312.3 (20:0 fatty acid)

LPC(20:1)

Precursor ion: [M+H]⁺ at m/z 550.3

Product ions: 184.1, 310.3 (20:1 fatty acid)

LPC(20:2)

Precursor ion: [M+H]⁺ at m/z 548.3

Product ions: 184.1, 308.3 (20:2 fatty acid)

LPC(20:3)

Precursor ion: [M+H]⁺ at m/z 546.3

Product ions: 184.1, 306.3 (20:3 fatty acid)

LPC(20:4)

Precursor ion: [M+H]⁺ at m/z 544.3

Product ions: 184.1, 304.3 (20:4 fatty acid)

LPC(20:5)

Precursor ion: [M+H]⁺ at m/z 542.3

Product ions: 184.1, 302.3 (20:5 fatty acid)

LPC(22:4)

Precursor ion: [M+H]⁺ at m/z 572.3

Product ions: 184.1, 332.3 (22:4 fatty acid)

LPC(22:5)

Precursor ion: [M+H]⁺ at m/z 570.3

Product ions: 184.1, 330.3 (22:5 fatty acid)

LPC(22:6)

Precursor ion: [M+H]⁺ at m/z 568.3

Product ions: 184.1, 328.3 (22:6 fatty acid)

2. Lysophosphatidylethanolamines (LPEs):

LPE(14:0)

Precursor ion: [M-H]⁻ at m/z 452.3

Product ions: 196.0 (Phosphoethanolamine head group), 226.3 (14:0 fatty acid)

LPE(16:0)

Precursor ion: [M-H]⁻ at m/z 480.3

Product ions: 196.0, 256.3 (16:0 fatty acid)

LPE(16:1)

Precursor ion: [M-H]⁻ at m/z 478.3

Product ions: 196.0, 254.3 (16:1 fatty acid)

LPE(18:0)

Precursor ion: [M-H]⁻ at m/z 508.3

Product ions: 196.0, 284.3 (18:0 fatty acid)

LPE(18:1)

Precursor ion: [M-H]⁻ at m/z 506.3

Product ions: 196.0, 282.3 (18:1 fatty acid)

LPE(18:2)

Precursor ion: [M-H]⁻ at m/z 504.3

Product ions: 196.0, 280.3 (18:2 fatty acid)

LPE(18:3)

Precursor ion: [M-H]⁻ at m/z 502.3

Product ions: 196.0, 278.3 (18:3 fatty acid)

LPE(20:0)

Precursor ion: [M-H]⁻ at m/z 536.3

Product ions: 196.0, 312.3 (20:0 fatty acid)

LPE(20:1)

Precursor ion: [M-H]⁻ at m/z 534.3

Product ions: 196.0, 310.3 (20:1 fatty acid)

LPE(20:2)

Precursor ion: [M-H]⁻ at m/z 532.3

Product ions: 196.0, 308.3 (20:2 fatty acid)

LPE(20:3)

Precursor ion: [M-H]⁻ at m/z 530.3

Product ions: 196.0, 306.3 (20:3 fatty acid)

LPE(20:4)

Precursor ion: [M-H]⁻ at m/z 528.3

Product ions: 196.0, 304.3 (20:4 fatty acid)

LPE(20:5)

Precursor ion: [M-H]⁻ at m/z 526.3

Product ions: 196.0, 302.3 (20:5 fatty acid)

LPE(22:4)

Precursor ion: [M-H]⁻ at m/z 558.3

Product ions: 196.0, 332.3 (22:4 fatty acid)

LPE(22:5)

Precursor ion: [M-H]⁻ at m/z 556.3

Product ions: 196.0, 330.3 (22:5 fatty acid)

LPE(22:6)

Precursor ion: [M-H]⁻ at m/z 554.3

Product ions: 196.0, 328.3 (22:6 fatty acid)

3. Phosphatidylcholines (PCs):

PC(14:0/14:0)

Precursor ion: [M+H]⁺ at m/z 662.5

Product ions: 184.1 (Phosphocholine head group), 226.3 (14:0 fatty acid)

PC(14:0/18:1)

Precursor ion: [M+H]⁺ at m/z 718.5

Product ions: 184.1, 256.3 (16:0 fatty acid), 281.3 (18:1 fatty acid)

PC(16:0/16:0)

Precursor ion: [M+H]⁺ at m/z 734.5

Product ions: 184.1, 256.3 (16:0 fatty acid)

PC(16:0/18:1)

Precursor ion: [M+H]⁺ at m/z 760.5

Product ions: 184.1, 256.3 (16:0 fatty acid), 282.3 (18:1 fatty acid)

PC(18:0/18:0)

Precursor ion: [M+H]⁺ at m/z 788.5

Product ions: 184.1, 284.3 (18:0 fatty acid)

PC(18:1/18:1)

Precursor ion: [M+H]⁺ at m/z 786.5

Product ions: 184.1, 282.3 (18:1 fatty acid)

PC(18:2/18:2)

Precursor ion: [M+H]⁺ at m/z 784.5

Product ions: 184.1, 280.3 (18:2 fatty acid)

PC(16:0/22:6)

Precursor ion: [M+H]⁺ at m/z 806.5

Product ions: 184.1, 256.3 (16:0 fatty acid), 328.3 (22:6 fatty acid)

PC(18:0/22:6)

Precursor ion: [M+H]⁺ at m/z 834.5

Product ions: 184.1, 284.3 (18:0 fatty acid), 328.3 (22:6 fatty acid)

PC(18:1/22:6)

Precursor ion: [M+H]⁺ at m/z 832.5

Product ions: 184.1, 282.3 (18:1 fatty acid), 328.3 (22:6 fatty acid)

4. Phosphatidylethanolamines (PEs):

PE(14:0/14:0)

Precursor ion: [M-H]⁻ at m/z 650.5

Product ions: 196.0 (Phosphoethanolamine head group), 226.3 (14:0 fatty acid)

PE(14:0/18:1)

Precursor ion: [M-H]⁻ at m/z 706.5

Product ions: 196.0, 256.3 (16:0 fatty acid), 281.3 (18:1 fatty acid)

PE(16:0/16:0)

Precursor ion: [M-H]⁻ at m/z 722.5

Product ions: 196.0, 256.3 (16:0 fatty acid)

PE(16:0/18:1)

Precursor ion: [M-H]⁻ at m/z 748.5

Product ions: 196.0, 256.3 (16:0 fatty acid), 282.3 (18:1 fatty acid)

PE(18:0/18:0)

Precursor ion: [M-H]⁻ at m/z 776.5

Product ions: 196.0, 284.3 (18:0 fatty acid)

PE(18:1/18:1)

Precursor ion: [M-H]⁻ at m/z 774.5

Product ions: 196.0, 282.3 (18:1 fatty acid)

PE(18:2/18:2)

Precursor ion: [M-H]⁻ at m/z 772.5

Product ions: 196.0, 280.3 (18:2 fatty acid)

PE(16:0/22:6)

Precursor ion: [M-H]⁻ at m/z 794.5

Product ions: 196.0, 256.3 (16:0 fatty acid), 328.3 (22:6 fatty acid)

PE(18:0/22:6)

Precursor ion: [M-H]⁻ at m/z 822.5

Product ions: 196.0, 284.3 (18:0 fatty acid), 328.3 (22:6 fatty acid)

PE(18:1/22:6)

Precursor ion: [M-H]⁻ at m/z 820.5

Product ions: 196.0, 282.3 (18:1 fatty acid), 328.3 (22:6 fatty acid)

5. Phosphatidylinositols (PIs):

PI(16:0/16:0)

Precursor ion: [M-H]⁻ at m/z 809.5

Product ions: 241.0 (Inositol phosphate head group), 256.3 (16:0 fatty acid)

PI(16:0/18:1)

Precursor ion: [M-H]⁻ at m/z 835.5

Product ions: 241.0, 256.3 (16:0 fatty acid), 282.3 (18:1 fatty acid)

PI(18:0/18:0)

Precursor ion: [M-H]⁻ at m/z 863.5

Product ions: 241.0, 284.3 (18:0 fatty acid)

PI(18:1/18:1)

Precursor ion: [M-H]⁻ at m/z 861.5

Product ions: 241.0, 282.3 (18:1 fatty acid)

PI(16:0/22:6)

Precursor ion: [M-H]⁻ at m/z 883.5

Product ions: 241.0, 256.3 (16:0 fatty acid), 328.3 (22:6 fatty acid)

PI(18:0/22:6)

Precursor ion: [M-H]⁻ at m/z 911.5

Product ions: 241.0, 284.3 (18:0 fatty acid), 328.3 (22:6 fatty acid)

PI(18:1/22:6)

Precursor ion: [M-H]⁻ at m/z 909.5

Product ions: 241.0, 282.3 (18:1 fatty acid), 328.3 (22:6 fatty acid)

6. Cholesterol Esters (CEs):

CE(18:1)

Precursor ion: [M+NH4]⁺ at m/z 666.6

Product ions: 369.3 (Cholesterol backbone), 265.3 (Fatty acid)

CE(20:4)

Precursor ion: [M+NH4]⁺ at m/z 670.6

Product ions: 369.3, 319.3 (20:4 fatty acid)

CE(22:6)

Precursor ion: [M+NH4]⁺ at m/z 696.6

Product ions: 369.3, 327.3 (22:6 fatty acid)

7. Sphingomyelins (SMs):

SM(16:0)

Precursor ion: [M+H]⁺ at m/z 703.6

Product ions: 184.1 (Phosphocholine head group), 283.3 (Fatty acid)

SM(18:1)

Precursor ion: [M+H]⁺ at m/z 731.6

Product ions: 184.1, 311.3 (Fatty acid)

SM(20:0)

Precursor ion: [M+H]⁺ at m/z 759.6

Product ions: 184.1, 339.3 (Fatty acid)

SM(24:1)

Precursor ion: [M+H]⁺ at m/z 813.7

Product ions: 184.1, 367.3 (Fatty acid)

8. Diacylglycerols (DAGs):

DAG(16:0/16:0)

Precursor ion: [M+NH4]⁺ at m/z 570.5

Product ions: 313.3 (Diacyl backbone), 256.3 (16:0 fatty acid)

DAG(16:0/18:1)

Precursor ion: [M+NH4]⁺ at m/z 596.5

Product ions: 313.3, 282.3 (18:1 fatty acid)

DAG(18:0/18:1)

Precursor ion: [M+NH4]⁺ at m/z 624.5

Product ions: 313.3, 284.3 (18:0 fatty acid)

DAG(18:1/18:1)

Precursor ion: [M+NH4]⁺ at m/z 622.5

Product ions: 313.3, 282.3 (18:1 fatty acid)

DAG(18:0/22:6)

Precursor ion: [M+NH4]⁺ at m/z 678.5

Product ions: 313.3, 328.3 (22:6 fatty acid)

9. Triacylglycerols (TAGs):

TAG(16:0/18:1/18:1)

Precursor ion: [M+NH4]⁺ at m/z 874.7

Product ions: 313.3 (Glycerol backbone), 256.3 (16:0 fatty acid), 282.3 (18:1 fatty acid)

TAG(18:0/18:1/18:2)

Precursor ion: [M+NH4]⁺ at m/z 900.7

Product ions: 313.3, 284.3 (18:0 fatty acid), 280.3 (18:2 fatty acid)

TAG(18:1/18:1/18:2)

Precursor ion: [M+NH4]⁺ at m/z 898.7

Product ions: 313.3, 282.3 (18:1 fatty acid), 280.3 (18:2 fatty acid)

TAG(16:0/18:1/22:6)

Precursor ion: [M+NH4]⁺ at m/z 938.7

Product ions: 313.3, 256.3 (16:0 fatty acid), 328.3 (22:6 fatty acid)
